# Cyclical adaptation of measles virus quasispecies to epithelial and lymphocytic cells: To V, or not to V

**DOI:** 10.1371/journal.ppat.1007605

**Published:** 2019-02-15

**Authors:** Ryan C. Donohue, Christian K. Pfaller, Roberto Cattaneo

**Affiliations:** 1 Department of Molecular Medicine, Mayo Clinic, Rochester, MN, United States of America; 2 Mayo Clinic Graduate School of Biomedical Sciences, Mayo Clinic, Rochester, MN, United States of America; Johns Hopkins Bloomberg School of Public Health, UNITED STATES

## Abstract

Measles virus (MeV) is dual-tropic: it replicates first in lymphatic tissues and then in epithelial cells. This switch in tropism raises the question of whether, and how, intra-host evolution occurs. Towards addressing this question, we adapted MeV either to lymphocytic (Granta-519) or epithelial (H358) cells. We also passaged it consecutively in both human cell lines. Since passaged MeV had different replication kinetics, we sought to investigate the underlying genetic mechanisms of growth differences by performing deep-sequencing analyses. Lymphocytic adaptation reproducibly resulted in accumulation of variants mapping within an 11-nucleotide sequence located in the middle of the phosphoprotein (P) gene. This sequence mediates polymerase slippage and addition of a pseudo-templated guanosine to the P mRNA. This form of co-transcriptional RNA editing results in expression of an interferon antagonist, named V, in place of a polymerase co-factor, named P. We show that lymphocytic-adapted MeV indeed produce minimal amounts of edited transcripts and V protein. In contrast, parental and epithelial-adapted MeV produce similar levels of edited and non-edited transcripts, and of V and P proteins. Raji, another lymphocytic cell line, also positively selects V-deficient MeV genomes. On the other hand, in epithelial cells V-competent MeV genomes rapidly out-compete the V-deficient variants. To characterize the mechanisms of genome re-equilibration we rescued four recombinant MeV carrying individual editing site-proximal mutations. Three mutations interfered with RNA editing, resulting in almost exclusive P protein expression. The fourth preserved RNA editing and a standard P-to-V protein expression ratio. However, it altered a histidine involved in Zn^2+^ binding, inactivating V function. Thus, the lymphocytic environment favors replication of V-deficient MeV, while the epithelial environment has the opposite effect, resulting in rapid and thorough cyclical quasispecies re-equilibration. Analogous processes may occur in natural infections with other dual-tropic RNA viruses.

## Author summary

Key questions in infectious disease are how pathogens adapt to different cells of their hosts, and how the interplay between the virus and host factors controls the outcome of infection. Human measles virus (MeV) and related animal morbilliviruses provide important models of pathogenesis because they are dual-tropic: they replicate first in immune cells for spread through the body, and then in epithelial cells for transmission. We sought here to define the underlying molecular and evolutionary processes that allow MeV to spread rapidly in either lymphocytic or epithelial cells. We discovered unexpectedly rapid and thorough genome adaptation to these two tissues. Genome variants that cannot express functional V protein, an innate immunity control protein, are rapidly selected in lymphocytic cells. These variants express only the P protein, a polymerase co-factor, instead of expressing P and V at similar levels. Upon passaging in epithelial cells, V-competent MeV genome variants rapidly re-gain dominance. These results suggest that cyclical quasispecies re-equilibration may occur in acute MeV infections of humans, and that suboptimal variants in one environment constitute a low frequency reservoir for adaptation to the other, where they become dominant.

## Introduction

RNA virus populations are quasispecies. Quasispecies, also known as mutant spectra, clouds or swarms, are genome distributions that are generated upon replication of RNA viruses in infected cells and organisms [1]. Quasispecies can adapt to dynamic environments and evade selective pressures exerted by antibodies or antiviral drugs [2]. Next-generation sequencing, which greatly expands the capacity to capture low frequency variants within virus quasispecies is beginning to reveal the mechanisms driving mutant spectra adaptation during some RNA virus infections, including those of HIV and HCV [3, 4]. Inter-host adaptation of RNA viruses, for example of arboviruses to arthropods and vertebrates, or of influenza viruses to birds and mammals, are well characterized [5, 6], but insights about genetic diversity and adaptation of viruses that replicate sequentially in two tissue niches of the same host are rare.

MeV provides an important model of pathogenesis due to its dual-tropic nature: it replicates first in lymphatic tissues and then in epithelial cells. Receptors determine MeV tropism [7]. After contagion, the signaling lymphocyte activation molecule (SLAM) [8] mediates MeV entry in alveolar macrophages and dendritic cells that ferry the infection through the airway epithelium and spread it to local lymph nodes [9]. MeV then spreads in immune tissues, causing immunosuppression [10, 11]. Immune cells deliver the infection to columnar epithelial cells that express nectin-4, the MeV epithelial receptor [12, 13]. Nectin-4 expression in the upper airway epithelia accounts for efficient MeV replication at a location facilitating extremely efficient contagion [7].

MeV is a negative strand RNA virus of the genus *Morbillivirus* in the family *Paramyxoviridae* [14]. Morbillivirus genomes are organized into six contiguous, non-overlapping transcription units separated by three untranscribed nucleotides and coding for eight viral proteins, in the order (positive strand): 5’-N-P/V/C-M-F-H-L-3’ [15]. The second transcription unit codes for two non-structural proteins, C and V, that are expressed in non-traditional ways. C is translated from an alternative reading frame accessed from a downstream internal start codon [16]. V is translated from mRNAs in which the viral polymerase inserts one pseudo-templated guanosine after a highly conserved poly-purine stretch, a process of co-transcriptional RNA editing that results in translation of a unique cysteine-rich 68-amino acid carboxyl-terminal domain [17]. Both V and C interfere with the host immune response [18–21].

MeV populations are quasispecies. An early analysis of infections of HeLa cells with a vaccine-lineage MeV estimated the intra-population diversity at 6–9 positions per genome [22]. However, this and many other studies of MeV biology are based on vaccine-lineage MeV strains that, during the attenuation process, were adapted for growth in stable cell lines of disparate origin. Insights about the quasispecies composition of a wild-type MeV replicating in lymphocytic or epithelial cells, the most relevant cell types for infection, are not available.

To address this gap in knowledge, we performed cell-specific adaptation studies. We discovered that MeV genomes that cannot express functional V protein are rapidly selected in lymphocytic cells. Upon passaging in epithelial cells, V-competent MeV genomes rapidly re-gain dominance, indicating that suboptimal variants in lymphocytes can serve as a low frequency reservoir of alleles for adaptation to epithelial cells.

## Results

### MeV adaptation to lymphocytic or epithelial cells

We sought to obtain virus inocula of consistent quasispecies composition, and to passage them in set cellular environments. Towards obtaining consistent inocula, we generated MeV from a cDNA copy of its genome, using standardized procedures based on the overlay of “rescue” 293-derived cells with Vero-hSLAM cells [23]. In particular, we operated with the infectious cDNA from the Ichinose-B (IC-B) strain that has been derived from a wild-type virus [24] and was extensively used for pathogenesis studies in primates [25–29]. To facilitate monitoring the progression of infection during passaging, we used a virus expressing a reporter protein, MeV-IC323-mCherry. We chose lymphocytic Granta-519 cells [30, 31] and lung epithelial H358 cells [12, 32] as model environments.

To assess whether MeV adapts to these environments, we passaged the MeV-IC323-mCherry inoculum (passage 1, p1) 14 times in either lymphocytic Granta-519 cells, or in airway epithelial H358 cells. We used a multiplicity of infection (MOI) of 0.1 for the first infection, followed by inoculations with 20% of the cell-associated inoculum for each subsequent infection. This strategy, while based on variable MOIs across passaging, allowed to maintain sufficient levels of infectious output (**S1 Fig,** top panel**)**. The passaged viruses were named L14 and E14, respectively.

**Fig 1:**
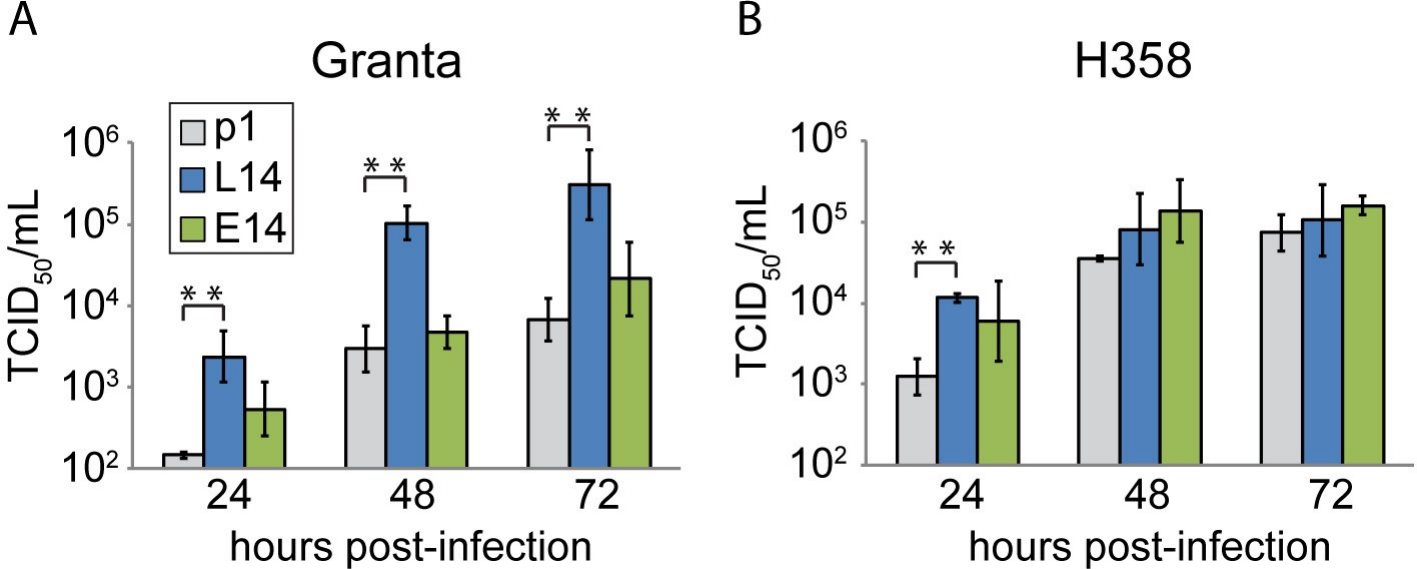
MeV passaged on a lymphocytic cell line has altered growth kinetics. (A and B) Titers of parental (p1) and passaged viruses on Granta-519 (A) and H358 (B) cells. p1 was passaged 14 times on lymphocytes (L14) or epithelial cells (E14). One million Granta or H358 cells were infected with either p1, L14, or E14 at MOI 0.03 and harvested at 24, 48, or 72 h post infection. Titers were determined using triplicate end point dilution assays on Vero hSLAM cells. Results are shown as means ±SD of n = 3. Asterisks indicate statistically significant differences compared to p1 (**, P<0.01; Student’s t-test).

An indication of adaptation to Granta cells came from a growth kinetics analysis of L14 in either Granta (**Fig 1A**) or H358 (**Fig 1B**) cells. In lymphocytic Granta cells, the L14 virus replicated to higher titers than either p1 or E14 (**Fig 1A**). In contrast, in H358 cells all three viruses reached similar titers (**Fig 1B**). These data suggest that significant changes in quasispecies composition may have occurred during passage on Granta cells.

### Editing site-proximal genomic variants are selected in lymphocytic cells

**Fig 2:**
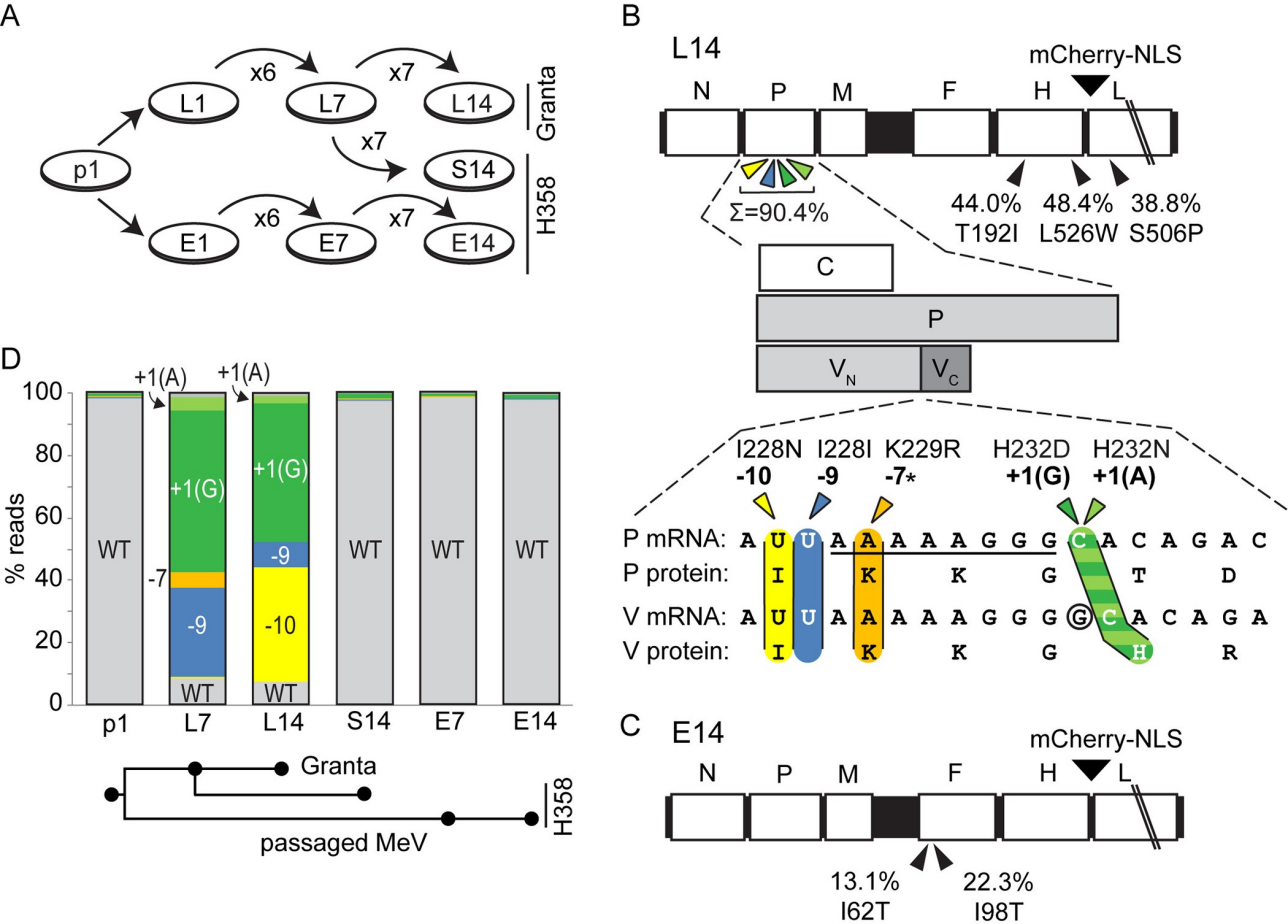
Different P gene editing site-proximal variants are selected in lymphocytic cells. (A) Strategy for MeV passaging. The original inoculum (p1) was passaged 14 times on Granta cells (L1 to L14) or H358 cells (E1 to E14). L7 was also passaged on H358 cells seven times to generate S14. (B) Genetic variants selected by adaptation to lymphocytic cells (L14). Coding regions on the MeV genome are shown by white boxes and non-coding regions are in black (top). Positions of cell-specific variants above 10% frequency not present in p1 are shown using a black arrowhead, and their allelic percentages and amino acid changes below. The four nearby variants in the P gene (colored) is shown as a sum. A schematic of P gene coding regions and locations of the lymphocytic variants are shown in the middle of panel B. C, generated from an internal AUG start site; P, generated from the first AUG unedited transcript; and V, generated from transcripts with an additional G inserted after the AAAAAGGG sequence (underlined). V shares the first 231 amino acids with P (V_N_), but has a different C-terminal domain (V_C_). Variants are indicated by colored arrowheads and their positions relative to the G insertion site (circled) are shown above the arrowheads. The -7 variant (orange) was only detected in passage L7, indicated by an asterisk. Amino acid sequences are shown below the nucleotide sequences. (C) Allelic variants selected after 14 passages in epithelial cells. Conventions as in panel B. (D) Analysis of editing site-proximal variants across passage history. The y-axis shows the percentage reads with the indicated alleles. Alleles are colored as in panel B: WT (grey), -10 (yellow), -9 (blue), -7 (orange), +1(G) (dark green), and +1(A) (light green). The passages analyzed are indicated on the horizontal axis, and the passage history is drawn schematically below it.

This experiment was part of a plan to document the kinetics of quasispecies adaptation to different cell types (**Fig 2A**). In addition to lymphocytic (L) and epithelial (E) adaptations, the plan included sequential (S) adaptation, as a model of MeV replication within a host. Sequential adaptation consists of 7 passages on lymphocytic cells, followed by 7 passages on epithelial cells. We sought to obtain sequence information on the inoculum (p1), lymphocytic-adapted virus (L7 and L14 passages), epithelial-adapted virus (E7 and E14 passages), and sequentially adapted virus (S14 passage).

To focus our analyses on MeV genomic RNAs, we purified encapsidated genomes (ribonucleocapsids, RNP) of p1, L7, L14, E7, E14 and S14 by isopycnic centrifugation. The sequence of the purified nucleic acids was then analyzed by RNAseq. We obtained 1.5–3 million MeV specific reads for each virus RNP preparation (**S2A Fig**). Average coverages exceeded 10,000 reads per nucleotide over the length of all six genomes, and coverage never dropped below 1,000 reads per nucleotide (**S2B Fig**). **S1 Table** lists all alleles in any sample that differ by >10% from the reference sequence.

We initially focused on L14 because its replication kinetics suggests adaptation. **Fig 2B** (top) visualizes all variants detected at >10% frequency that were expanded selectively in lymphocytic cells. Strikingly, several of these variants are located near the middle of the P gene (**Fig 2B**, colored arrows) surrounding the G-insertion site. In particular, substitutions at positions -10, -9, -7, and +1, as numbered from the G-insertion site (position 2499 on the MeV genome), were represented at a higher level in the genomic population (**Fig 2B**, bottom). At position +1, two different variants were enriched: +1(A) and +1(G). Altogether, the five editing-proximal variants accounted for about 90% of the reads covering this site (**Fig 2B**, top). Notably, no read with more than one of these mutations was detected. This suggests that five different genomes with a single point mutation near the editing site were positively selected during lymphocytic passaging. In contrast, in epithelial-adapted virus E14, no editing site-proximal variants, but two variants present at >10% frequency were detected (**Fig 2C**). Both variants resulted in F protein amino acid changes. In addition, three non-cell-specific M gene variants were detected at different levels in all six analyses (**S1 Table**).

We then analyzed the evolution of the editing site-proximal sequences during quasispecies adaptation. We noted that in L7 and L14, the total editing-proximal variant pool accounted for about 90% of the genomes (**Fig 2D**). However, the pool composition changed: the -10 variant displaced the -9 variant between passages 7 and 14; the -7 variant, present at low levels in L7, faded into background by passage 14; yet the +1(G) variant remained relatively constant. On the other hand, no mutations surrounding the editing site were selected in epithelial-adapted MeV (**Fig 2D**, E7 and E14). Strikingly, re-adaptation to epithelial cells resulted in elimination of the editing-proximal variants (**Fig 2D**, S14). These data suggest that opposing selective pressures are exerted on RNA editing in lymphocytic and epithelial cells.

### RNA editing efficiency is reduced after passage in lymphocytic cells

**Fig 3:**
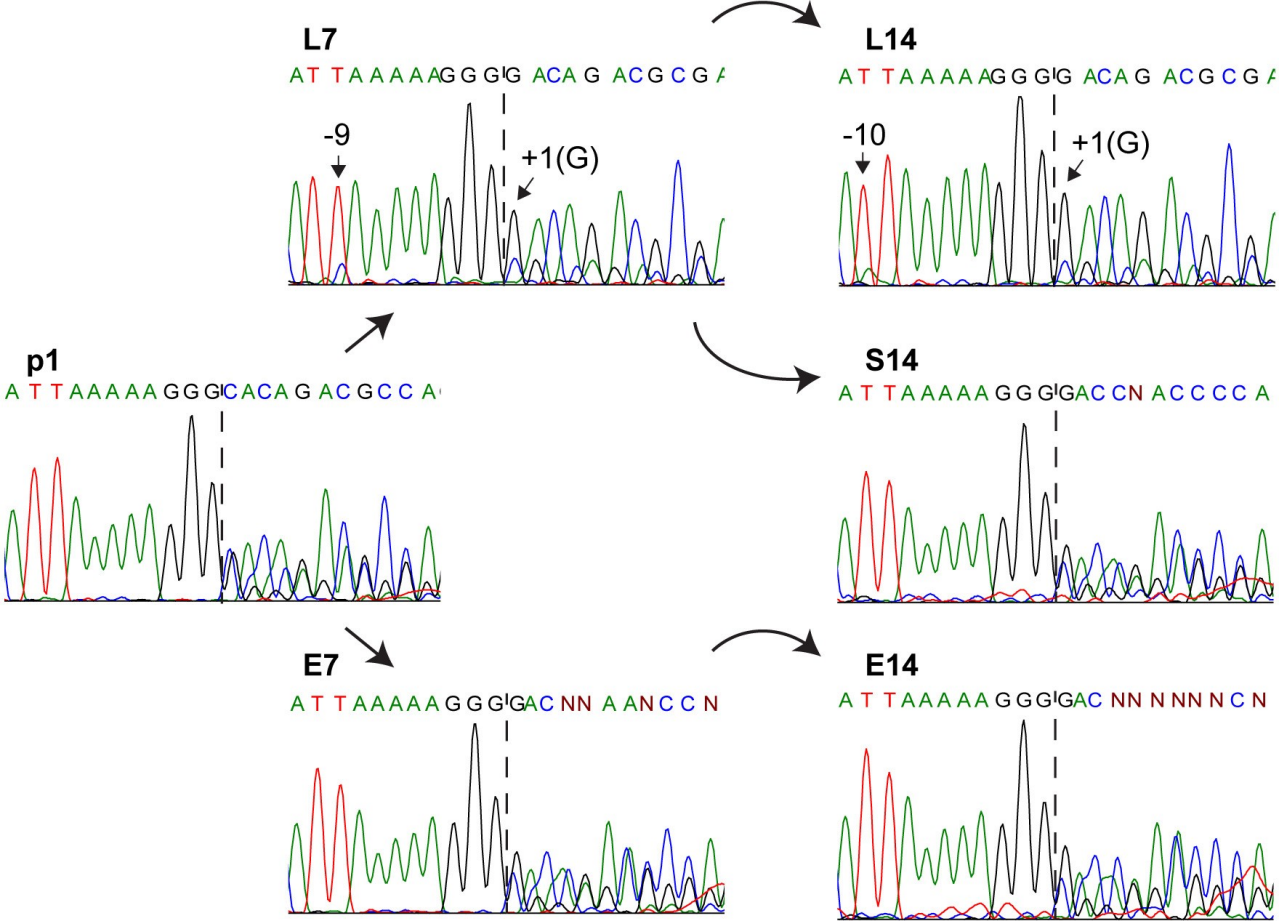
Passaging in lymphocytic cells selects against efficient RNA editing. Messenger sequences near the P gene editing site from different passages. Consensus sequences are displayed above the chromatograms. Passages are indicated above consensus sequences. Dashed line denotes the G insertion site. Mutations identified from deep sequencing data are indicated with small black arrows.

We then sought to assess whether the editing site-proximal mutations impacted the efficiency of G-nucleotide insertion in P mRNA. Towards this, we infected HeLa-hSLAM cells with p1, L7, L14, E7, E14 and S14 viruses, purified their mRNAs, amplified the relevant P gene segment, and performed dideoxy-sequencing. **Fig 3** (left panel) shows the p1 analysis: a homogeneous sequence extending over the conserved poly-purine tract AAAAAGGG becomes heterogeneous after the G-insertion position (vertical dotted line). The similar height of the G- and C-signals at this position indicates an approximately 1:1 ratio of edited (G) and unedited (C) transcripts.

In contrast, in lymphocytic cell-passaged viruses (L7 and L14), editing efficiency was strongly reduced. In **Fig 3** (upper two panels), the sequence of L7 shows heterogeneity at positions -9 and +1, as expected from the corresponding RNAseq data, and reduced RNA editing, indicated by the smaller secondary peaks from position +2 onwards. Similarly, the sequence of L14 shows the expected -10 and +1 heterogeneity, and reduced RNA editing. Since the +1 variants are downstream of the G insertion site, which complicates interpretation of the chromatograms, we performed a complementary analysis with a reverse primer that confirmed the above conclusions (**S3 Fig,** top three panels).

Contrastingly, in epithelial cell-passaged viruses editing efficiency remained near 50% (**Fig 3**, bottom two panels E7 and E14). Re-adaptation of L7 virus to epithelial cells restored efficient RNA editing (**Fig 3**, middle row, panel S14). These data directly confirm that opposing selective pressures are exerted on RNA editing in lymphocytic and epithelial cells.

### Lymphocytic cell-adapted viruses express less V protein

**Fig 4:**
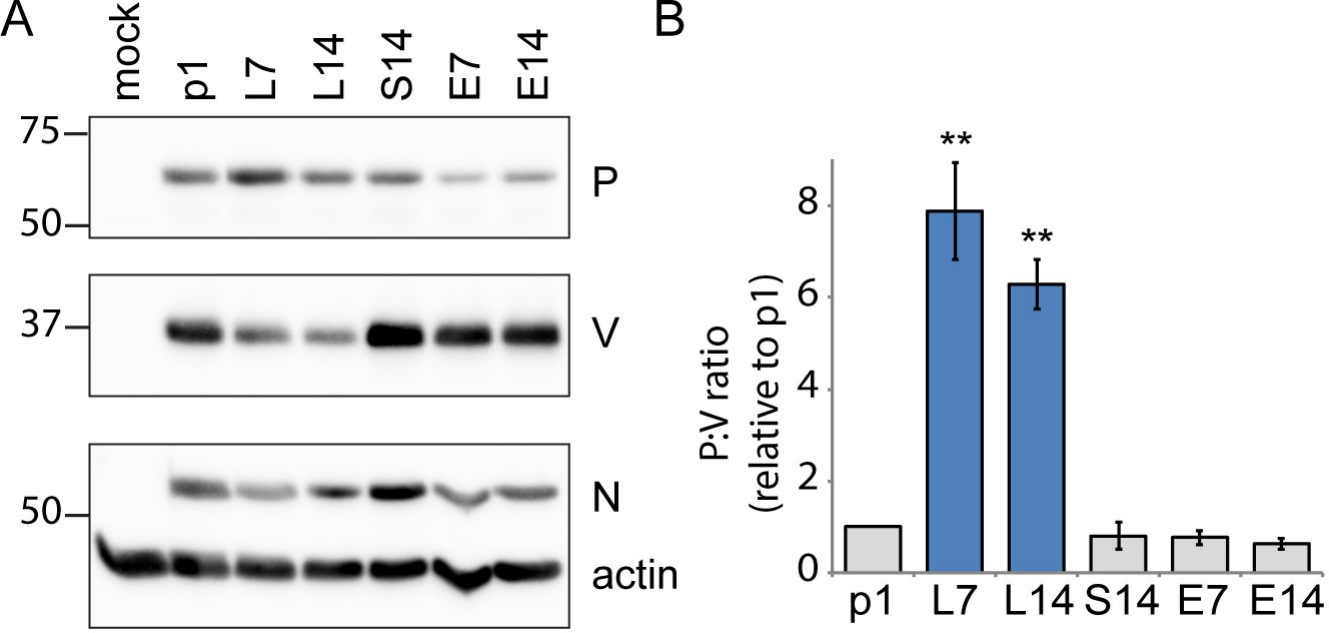
Passaging in lymphocytic cells selects against V protein expression. (A) Western blot analysis of viral protein expression 48 h post-infection. Lysates from HeLa-hSLAM cell infections (MOI 0.1) with the viruses indicated were probed with antisera against the P, V, or N proteins or cellular actin, as indicated on the right. Actin was a loading control and N an infection control. Molecular weight markers (kDa) are indicated on the left. (B) Quantification of western blot results. Band intensities were measured for P and V. The P-to-V ratio calculated for p1 was set to 1. The P-to-V ratios of passaged viruses are shown after adjustment with respect to p1. Results are shown as mean ±SD of n = 3. Asterisks indicate statistically significant differences compared to p1 (**, P<0.01; Student’s t-test).

We then assessed whether reduced P mRNA editing negatively impacts V protein expression. Towards this, we infected cells with p1, L7, L14, E7, E14 and S14 viruses, extracted their proteins, and estimated the relative abundance of P and V by immunoblot. The analyses of **Fig 4A** show that in p1 the V signal was stronger than the P signal. In contrast, the V and P signals in L7 and L14 were of similar intensities. The V signal of the S14 virus, which was re-adapted to epithelial cells, was stronger than the P signal, as were the V signals of the viruses exclusively passaged on epithelial cells. **Fig 4B** shows quantification of signal strength in three repeat experiments. The P:V ratio of p1 was set at 1. This ratio was between 6 and 8 for L7 and L14, while there was no statistical significance between p1 and the epithelial-adapted or sequentially passaged MeV. Thus, lymphocytic cell-adapted viruses express proportionately less V protein.

### Selection of V-deficient MeV variants is fast and reproducible

To assess whether selection of V-deficient MeV variants is reproducible, we infected the same lymphocytic (Granta) and epithelial (H358) cell lines with MeV-IC323-mCherry-uN. This virus expresses the two polymerase subunits P and L in the same ratio as the parental, non-recombinant, MeV because the added transcription unit with mCherry is inserted upstream of the N gene, rather than downstream of H. As in the first experiment, we passaged this virus either 14 times on lymphocytic cells, or 14 times on epithelial cells at an initial MOI of 0.1 This was followed by inoculations with a fraction (10%) of the cell-associated inoculum for each subsequent infection, which allowed to maintain sufficient levels of infectious output (**S1 Fig,** bottom panel). We then performed RNAseq on purified viral RNP, and identified genomic variants represented at or above the 10% level in any passage examined (**S2 Table**).

**Fig 5:**
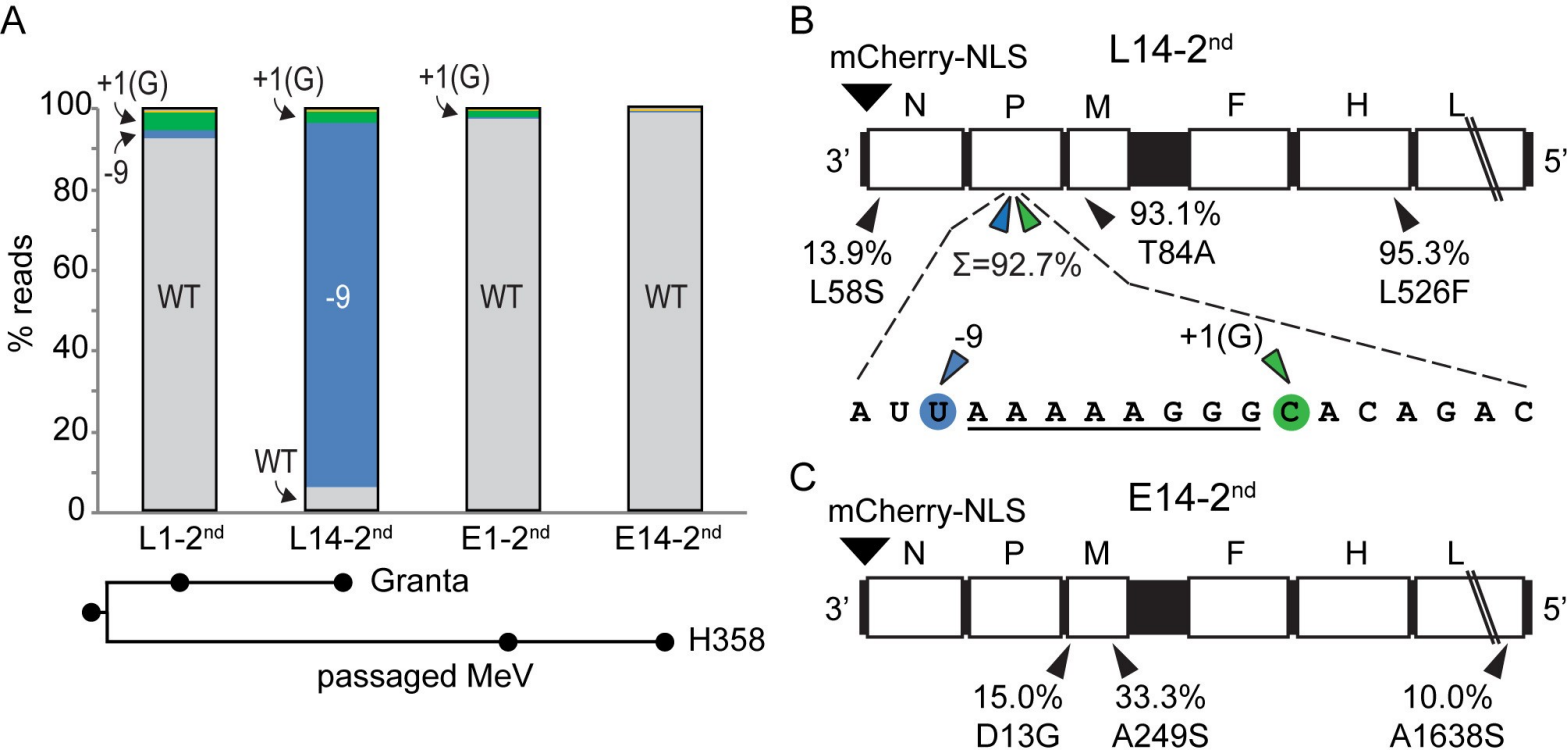
Editing site-proximal variants are consistently selected in lymphocytic cells. (A) Variants are color coded as in Fig 2. The passages analyzed are indicated on the horizontal axis. A schematic of the passage history is indicated below. (B) Genome-wide variants selected by adaptation to lymphocytic cells (L14-2^nd^). Coding regions are shown in white boxes and non-coding regions in black with the mCherry position indicated above the genome. Variants above 10% as compared to the reference genome are indicated below the genome with their percentage and predicted amino acid changes (arrowheads). (C) Genome-wide variants selected by adaptation to epithelial (E14-2^nd^) cells. Conventions as in panel B.

**Fig 5A** illustrates the results of this passaging experiment. This time we examined viral genomes at an early time point after passage 1 (L1-2^nd^ or E1-2^nd^) and again after passage 14 (L14-2^nd^ or E14-2^nd^). In passage L1-2^nd^, we noted a significant over-representation of the +1(G) substitution (4.4% allelic frequency), and of the -9 variant (2.1% allelic frequency). At the end of lymphocytic selection (L14-2^nd^), the -9 variant approached 90% of the population, and in combination the editing site-proximal variants accounted for nearly 93% of the population. In contrast, after either 1 or 14 epithelial passages (E14-2^nd^), editing site-proximal variants accounted for about 2% and less than 1% of the population, respectively.

Other mutations were selected in the repeat passaging experiment. In L14-2^nd^, one M gene variant and the H gene variant causing the amino acid change L526F were found in >90% of the reads, and one N gene variant in about 14% of the reads (**Fig 5B**). In contrast, in E14-2^nd^ two M and one L gene variants accumulated in 10–30% of the reads. Since these mutations were not enriched in the first passaging experiment, they could be adventitious early events that have no impact on adaptation. On the other hand, in the first experiment the H protein variant L526W emerged, suggesting reproducible selective advantage for an aromatic residue at H position 526. Interestingly, L526 is adjacent to a hydrophobic groove relevant for receptor interactions [33].

In combination, the results of the first and second experiment indicate that the key adaptation to the Granta cell environment is the selection of V-deficient mutants. Selection is fast, and independent of the ratio of P and L protein expression. On the other hand, different types of V-deficient genomes are selected in repeat experiments.

### V restriction occurs in a second lymphocytic cell line

**Fig 6:**
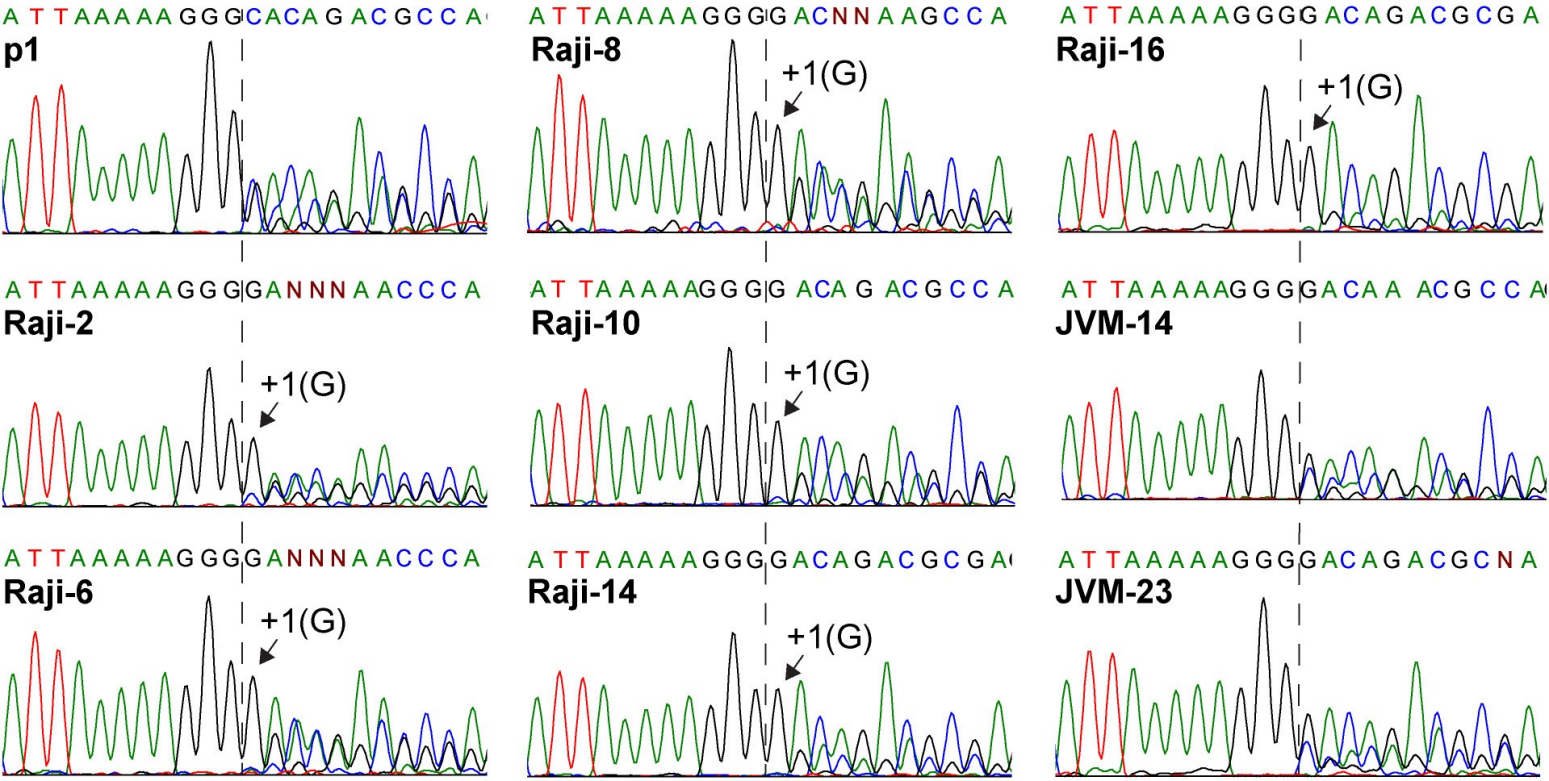
Editing-defective MeV are selected in Raji cells. Chromatograms of dideoxy-sequencing over the P gene editing site from viruses passaged on Raji or JVM-2 cells. Cell type used and passage numbers are indicated above the chromatograms. The +1(G) variant takes over the consensus (indicated above the chromatograms) from Raji passage 2 onwards. Secondary peaks downstream of the G-insertion site (dotted vertical line) reflect RNA editing efficiency. Late passages from JVM-2 cells indicate no reduced editing with no detectable proximal variants.

To assess whether V-restriction occurs in more than one cell line, we passaged the same virus used for the first Granta cells adaptation experiment in other lymphocytic cell lines, Raji and JVM-2. After two passages in Raji cells the +1(G) variant became dominant, and by passage 6 the wild-type C allele faded into background (**Fig 6**). A complementary analysis with a reverse primer confirmed this conclusion (**S3 Fig**, bottom panel). Interestingly, editing efficiency steadily decreased over time, but lagged selection of +1(G) (**Fig 6**). This indicates that editing efficiency is not uniquely determined by the +1(G) mutation. Dideoxy-sequencing confirmed that the P gene mRNA of these passages was identical to the reference sequence and no detectable minor alleles were present. Conversely, RNA editing efficiency remained constant after MeV passaging in JVM-2 cells, even after 14 or 23 passages (**Fig 6**). Thus, V restriction occurs in at least two lymphocytic cell lines.

### V restriction occurs through different mechanisms

**Fig 7:**
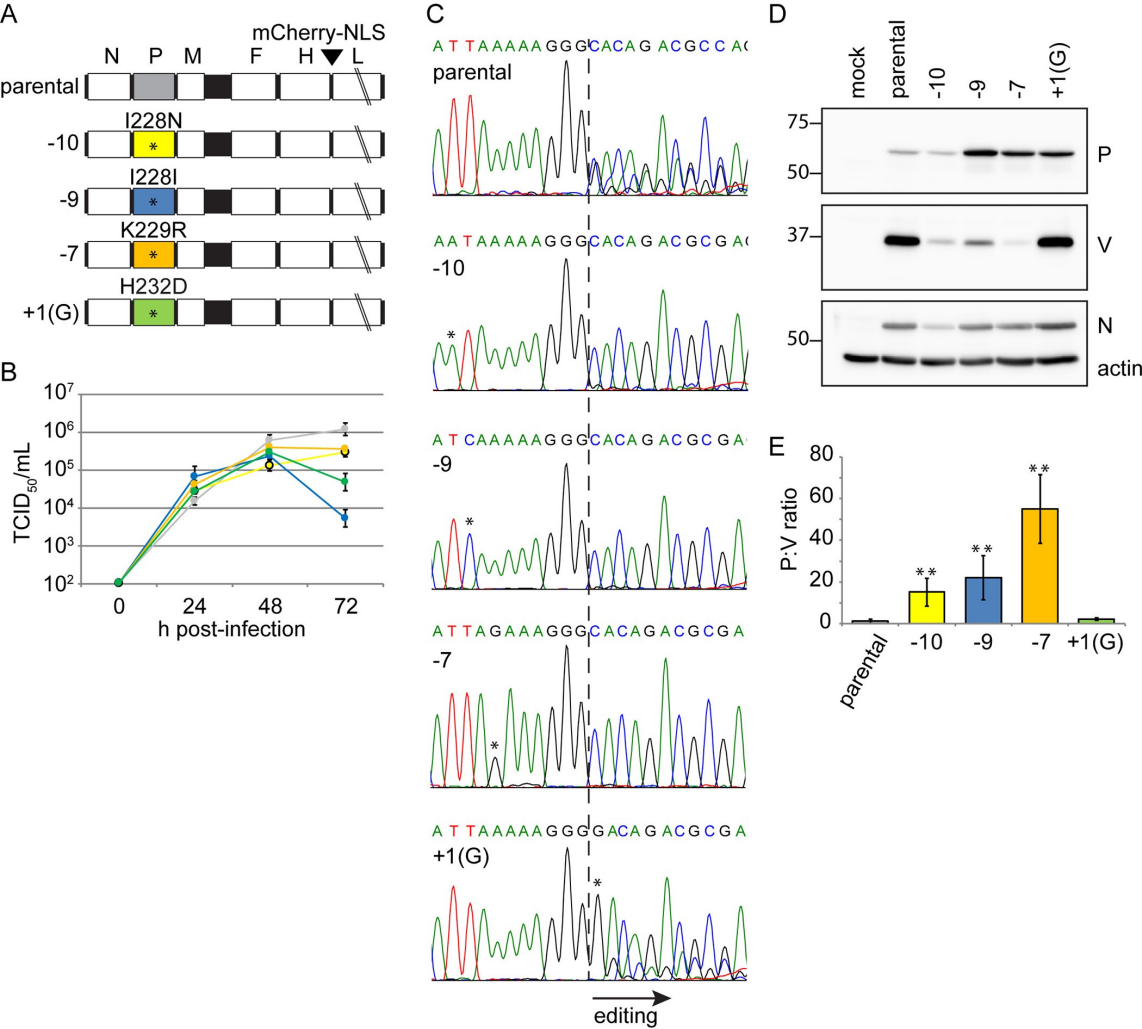
Recombinant MeVs with editing site-proximal variants are V-defective. (A) Genomes of the recombinant MeVs with single P gene substitutions. The numbers on the left of each genome indicate the position of the nucleotide changes relative to the G-insertion site, and the corresponding amino acid sequences are indicated above the P gene. (B) Growth curves of the recombinant viruses on Vero-hSLAM cells. Colors correspond to panel A. Values are shown as the mean of three replicates ± SD. (C) Analysis of RNA editing by the recombinant viruses after infection of HeLa-hSLAM cells 48 h post infection at MOI 0.1. Vertical lines within the chromatograms indicate the G-insertion site. Asterisks above nucleotides indicate the position of the variant nucleotides. Secondary peaks downstream of the G-insertion site reflect the efficiency of RNA editing. (D) Proteins expressed by the recombinant viruses. Westerns blots were performed using lysates from the same infections as in (C), with sera against the N, P, and V proteins. Actin was used as a loading control. Size markers are indicated on the left of each panel. (E) Quantitation of protein expression levels. Values shown as mean of n = 3, ± SD. Calculations were normalized to the p1 ratio of P or V to N. Asterisks indicate statistically significant differences compared to p1 (**, P<0.01, Student’s t-test).

To assess by which mechanisms the editing site-proximal variants impact V expression, we generated viruses differing from parental MeV-IC323-mCherry by a single base. The genomes of these viruses bear the following substitutions: -10, -9, -7 and +1(G) (**Fig 7A**, top to bottom). We note that the +1(G) virus does not contain a G insertion, rather a C-to-G substitution at position 2499, which is the “+1” position, counting from the editing site. Three of these mutations result in the amino acid changes listed above the corresponding P genes, whereas the -9 mutation is silent.

We then assessed whether the four recombinant viruses have similar characteristics as the parental virus with a growth assay based on permissive Vero cells expressing the MeV receptor SLAM (Vero-hSLAM). **Fig 7B** indicates that, while the editing site-proximal variants grow to higher titers than wild type at an earlier time point, the wild-type MeV eventually reaches the highest titers.

We then assessed whether each mutation impacts RNA editing, and to which extent. As done previously with the passaged virus mixtures, RNA was extracted from HeLa-hSLAM cells infected with the editing-site proximal viruses, the relevant P gene segment amplified, and dideoxy-sequencing performed. The chromatograms of **Fig 7C** (second to fourth panel from top) indicate that in mutants -10, -9, and -7, RNA editing was not detectable (no secondary peaks after the dotted line). In contrast, in mutant +1(G) (bottom panel) editing was only slightly reduced as compared to the parental virus (top panel).

To assess whether mutations elsewhere in the genome could impact editing, we generated a recombinant virus with one standard and one mutated P gene (**S4 Fig**, top drawing). We then determined the editing efficiency of both P gene copies. We confirmed that only the mutated P gene copy had reduced editing capacity (**S4 Fig**, bottom). Thus, editing site-proximal variants directly govern editing efficiency.

We also characterized the impact of each mutation on V protein expression. For this, proteins were extracted from HeLa-hSLAM cells infected with the four viruses and expression levels of P and V compared to those of the viral N protein, and with cellular actin. The immunoblots of **Fig 7D** indicate that in mutants -10, -9, and -7, V protein expression was strongly reduced compared with that of the parental virus, whereas in mutant +1(G) V protein expression was maintained. We then quantified signal strength in three repeat experiments (**Fig 7E**). Relative to the parental P-to-V expression ratio, the -10, -9, -7, and +1(G) expression ratios were 15, 22, 55 and 2, respectively. These results are consistent with the levels of V mRNA expressed by the respective viruses.

These results indicate that the +1(G) virus edits P mRNAs and expresses V protein efficiently. However, the corresponding H232D mutation may inactivate V protein function: histidine 232, which together with three cysteines coordinates a Zn^2+^ ion, is essential for innate immunity interference by the V protein of MeV and other paramyxoviruses [34].

### Rapid dilution of V-deficient genomes in epithelial cells

Both lymphocytic adaptation experiments yielded 10-to-1 mixtures of V-deficient and V-competent genomes. To model what may occur when genome mixtures are transferred to host epithelial cells, we performed a competition assay. We inoculated H358 cells with an excess of V-deficient virus mixed with either 1%, 3%, 10% or 30% V-competent wild-type virus at a MOI of 0.1. The -9 variant was used as a V-deficient genome model. We followed how the two genomes competed by purifying mRNA from infected cells, amplifying a P gene segment, and performing dideoxy-sequencing.

**Fig 8:**
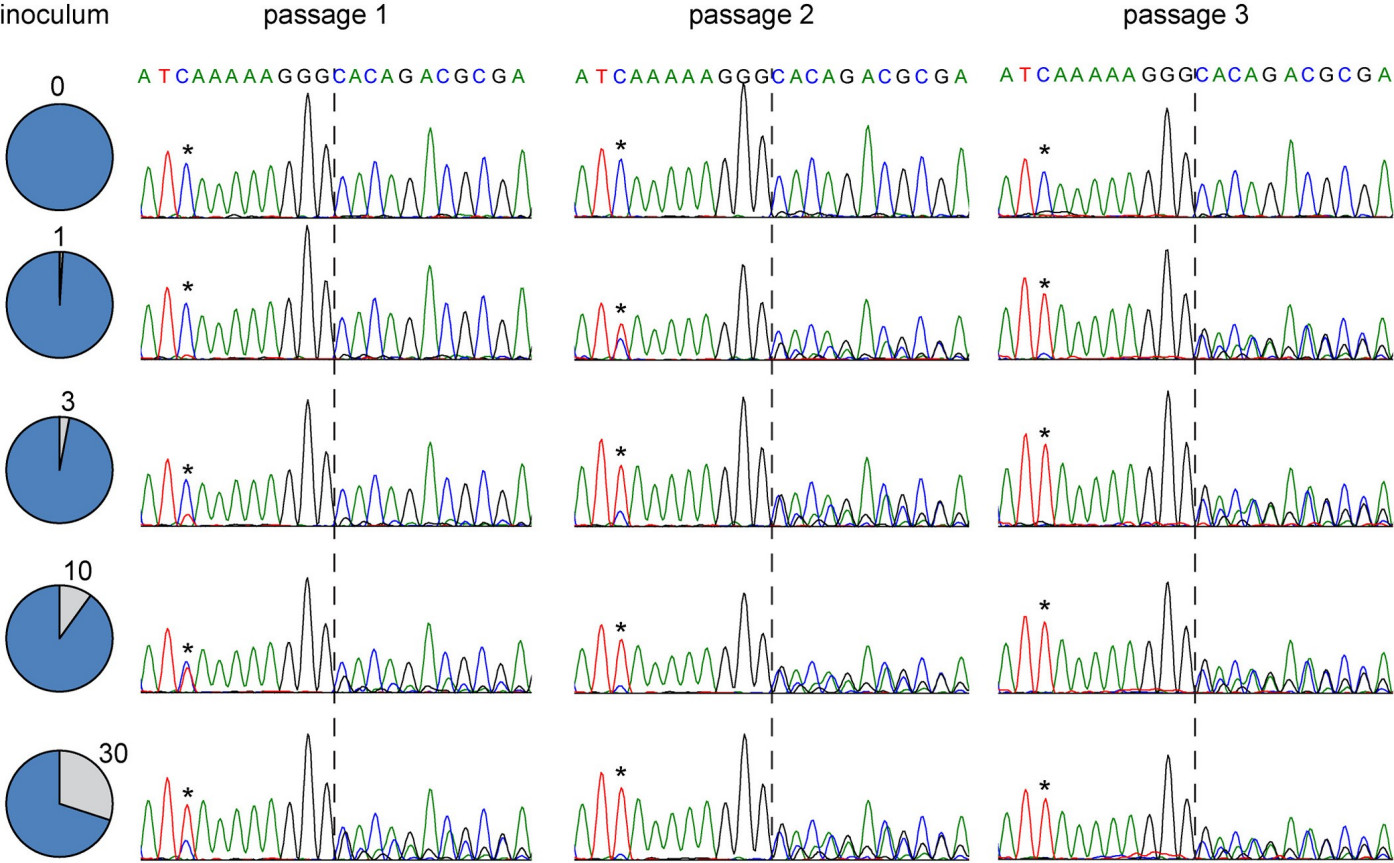
Rapid dilution of V-restricted genomes in epithelial cells. Pie charts on the left illustrate mixing experiments in which -9 V-deficient MeV (blue) were spiked with wild-type V-competent MeV (grey). The percentage of wild-type MeV is indicated. Chromatograms of P gene sequences are shown after passages 1 to 3 in H358 cells. Asterisks indicate -9 allele positions. Dotted line indicates the G-insertion site.

We compared mRNA frequencies after 1, 2, or 3 passages of V-deficient variants either alone (top chromatograms), or mixed with increasing amounts of wild-type genomes (second to fifth row) (**Fig 8**). The relative amounts of the genomes are proportional to the heights of the C (V-deficient) or T (wild type) signals at position -9 (asterisks). After one passage, the fraction of wild-type genomes in the four mixtures increased, reaching about 10%, 25%, 50% and 70%, respectively (**Fig 8**, left column). At passage 2, V-deficient genomes were in the minority in all mixtures (**Fig 8**, right column). Consistently, at passage 2 secondary peaks due to RNA editing were prominent in all mixtures (positions downstream of dotted line). At passage 3, wild-type genomes constituted more than 90% of the population even in the lowest initial dilution (1%). On the other hand, in pure V-deficient virus infections neither at passage 3 (top row, right column) nor at passage 7 (not shown), V-competent genomes were detected. Thus, provided that they constitute at least 1% of the initial population, V-competent genomes rapidly out-compete V-deficient genomes in H358 epithelial cells. Taken together, these data demonstrate that the sequential adaptation of the MeV genome to lymphocytic and epithelial cell lines results in cyclical selection of V-deficient and V-competent genomes.

## Discussion

Seeking to characterize processes that facilitate MeV adaptation to its two cellular environments, we discovered fast and thorough quasispecies re-equilibration. Our observations beg the question of why such a striking phenomenon was not previously described. Previous studies of tropism have mainly been focused on the attachment protein, rather than being approached with an unbiased genetic method. Another important consideration is that the standard procedure for MeV isolation relies on nasal secretions, and thus yields virus of epithelial origin. These viruses are isolated and grown on interferon-defective Vero cells expressing the primary MeV receptor human SLAM [35], the same cells used to grow our viruses. In this cellular environment, the standard “wild-type” MeV genome sequence is indeed dominant.

We asked what may happen in the initial phases of a host infection, when MeV replicates in lymphocytic tissue. To model this phase, we infected lymphocytic cell lines Granta or Raji and observed selection of MeV genome variants that cannot express functional V protein. These V-deficient genomes arise based on several different point mutations and after a few passages account for 90% of MeV genomes. Upon passaging in epithelial cells, V-competent wild-type genomes rapidly out-compete the V-deficient variants, and the quasispecies composition returns to the original equilibrium. Thus, in our experimental system V-competent genomes, which are sub-optimal variants in lymphocytes, constitute a low frequency variant pool for adaptation to epithelial cells.

Quasispecies re-equilibration is based on differential V protein expression. V proteins, which are conserved among most *Paramyxoviridae*, are polyvalent innate immunity controllers. The MeV V protein reduces both type I interferon signaling by inactivating STAT1 and STAT2 [18, 28, 36] and interferon production by inhibiting the cytoplasmic RNA sensor MDA5 [34, 37, 38]. While the residues interacting with STAT1 are located in the shared P and V amino-terminal half [39], those interacting with STAT2 and MDA5 are located in the V-unique carboxyl-terminal domain [21, 34]. Thus, V-deficient MeV are unable to control either STAT2-dependent or MDA5-dependent interferon activation.

Complete V-deficiency would be perplexing, even considering that Granta and Raji cells have reduced antiviral innate defenses [30, 40, 41]. However, the quasispecies growing in Granta cells include about 10% V-competent genomes. Thus, “just right” levels of V-protein expression may be required for efficient MeV spread in lymphocytic cells. Reduced innate immune defenses cannot be the only determinant of V-restriction because Vero-hSLAM cells do not select for V-defective mutants [42] while being interferon-defective [43].

In three of the four recombinant MeV with editing site-proximal mutations, RNA editing was minimal, and P protein expression enhanced at the expense of V protein expression. The exception was the +1(G) recombinant virus, which maintained near wild type RNA editing, and standard P-to-V protein expression ratio. However, the +1(G) mutation, which is silent for P, for V alters a histidine involved in Zn^2+^ binding [34]. This interferes with V-protein interactions with both STAT2 and MDA5 [34]. Positive selection of this mutant, which during the first experiment accounted for about half of the V-deficient genomes, suggests that inactivation of V protein function, rather than enhanced P protein expression, is key for adaptation to lymphocytic cells.

While editing efficiency is in the 30–50% range in different MeV strains propagated on Vero-hSLAM cells [42], a recent study revealed 5–20% editing efficiency in MeV mRNA extracted from brain autopsy materials of seven subacute sclerosing panencephalitis cases [44]. This indicates that during infection of humans editing efficiency can vary. It could also reflect rapid quasispecies adaptation to the neuronal environment.

Rapid adaptation to Granta and Raji cells could be explained by a negative effect of V on MeV replication in lymphocytic environments. The MeV V protein can limit viral RNA synthesis [45, 46], and a cellular co-factor differentially expressed in epithelial and lymphocytic cells could regulate this effect. Alternatively, the V-protein interaction with either STAT2, or MDA5, may be key for adaptation to lymphocytic cells. This hypothesis can be tested through the generation of selectively STAT2- or MDA5-blind MeV [28], or by passaging MeV in Granta cells that do not express STAT2 or MDA5 [47].

Rapid quasispecies re-equilibration may occur during acute MeV infections. This would be facilitated by the mode of MeV spread within hosts, which is based on the intercellular transfer of multiple genomes [48–50]. Experimental primate infections indicate that MeV spreads primarily through infected cells within a host [51]. In particular, MeV can spread between lymphatic cells through the formation of synapse-like interfaces [52] that are likely to transfer large numbers of genomes. Simultaneous transfer of genome packets may also occur in airway epithelia, based on the formation of intercellular pores [49]. Moreover, infectious MeV particles contain multiple genomes [48]. Thus, genome mixtures, rather than individual genomes, may spread through the host.

In conclusion, genomic adaptation of a dual-tropic RNA virus to its two natural cellular environments is unexpectedly rapid and thorough. Similar cyclical quasispecies re-equilibration processes may occur during natural infections with other dual-tropic RNA viruses. These include noroviruses, which infect epithelial and non-epithelial cell types [53], and HIV, which infects T-cells and macrophages. We suggest that the virulence of these dual-tropic RNA viruses may reflect the combined activity of distinct cell-specific quasispecies.

## Materials and methods

### Cells

Vero-hSLAM (kindly provided by Y. Yanagi,[35]), 293-4-46 [23], and HeLa-hSLAM [54] cells were cultivated in Dulbecco's high-glucose modified Eagle's medium (D-MEM; HyClone; GE Healthcare Life Sciences, Logan, UT) supplemented with 10% (vol/vol) fetal bovine serum (FBS; Gibco; Life Technologies, Carlsbad, CA), and 1% Penicillin-Streptomycin Solution (Pen-Strep; Corning; Tewksbury, MA). Vero-hSLAM and 293-4-46 cells were grown in the presence of Geneticin (G418; Corning; Fisher Scientific; Hampton, NH) at final concentrations of 0.5 mg/ml and 1.2 mg/ml, respectively. HeLa-hSLAM cells were grown in 0.1 mg/ml Zeocin (Gibco; Invitrogen; Carlsbad, CA). The mantle cell lymphoma cell lines Granta-519 [55, 56] (Cat. # ACC 342; DSMZ; Braunschweig, Germany) and JVM-2 [57] (Cat # CRL-3002; ATCC; Manassas, Virginia), the Burkitt’s lymphoma cell line Raji [58] (Cat # CCL-86; ATCC) and the bronchioalveolar carcinoma cell line H358 [59] (Cat. # CRL-5807; ATCC) were cultivated in RPMI 1640 (HyClone) supplemented with 10% (vol/vol) FBS and Pen-Strep.

### Generation of viruses used for adaptation studies

Recombinant MeV constructs were generated in the IC323 background (similar to the wild-type IC-B strain [24]). p(+)MV323(mCherryNLS)uN and p(+)MV323(mCherryNLS)H contain an additional transcription unit with mCherry fused to a triple repeat nuclear localization signal (NLS) either upstream of N or downstream of H, respectively. p(+)MV323(mCherryNLS)H was generated by transferring mCherry-NLS from pB(+)MVvac2(mCherryNLS)H [31] into p(+)MV323(GFP)H, replacing GFP, using the restriction sites MluI and AatII. Rescued MeV generated from p(+)MV323(mCherryNLS)H (named MeV-IC323-mCherry) was used for the first passaging experiment, and MeV from p(+)MV323(mCherryNLS)uN (named MeV-IC323-mCherry-uN) was used for the second passaging experiment.

### Generation of viruses with lymphocytic-selected mutations

p(+)MV323-eGFP-P(-9) was constructed as described previously (Singh et al, submitted). The editing site was modified in the -9 position (T to C) using complementary primers spanning CCAGCACTTCCGAGACACCCATCAAAAAGGGCACAGACGCGAGAT (mutagenized nucleotide is underlined) on the intermediate pCG-eGFP-P_323_ plasmid. The mutagenized eGFP-P_323_ construct was then cloned into an additional transcription unit of p(+)MV323(GFP)H downstream of H using MluI and AatII sites, replacing eGFP to generate the final construct.

MeV-IC323-P(-10)-mCherry (referred simply as mutant -10), and the (-9), (-7) and +1(G) mutants were generated first by performing site directed mutagenesis on the P gene plasmid, pCGPmeI-MVwtIC323-PmeI. Complementary primers used for mutagenesis span the follow sequence: CCAGCACTTCCGAGACACCCATTAAAAAGGGCACAGACGCGAGAT; which contained single mutated nucleotides (underlined) for each of the four viruses (T to A for -10, T to C for -9, A to G for -7, and C to G for +1G). The mutagenized pCG-Pmel-MVwtIC323-Pmel plasmids were then ligated into p(+)MV323 [24] using BstEI and BssHI restriction sites. An additional transcription unit containing an mCherry-NLS reporter was inserted downstream of H using MluI and AatII, generating p(+)MV323(-10)(mCherryNLS)H, p(+)MV323(-9)(mCherryNLS)H, p(+)MV323(-7)(mCherryNLS)H, and p(+)MV323(+1G)(mCherryNLS)H. All plasmids were verified using dideoxy-methods.

### Virus rescue, stock production, and passaging

Recombinant viruses were produced as reported previously [23], generating passage 0 (p0) stocks. These stocks are amplified from a single syncytium to a 10 cm^2^ plate with 5x10^6^ Vero-hSLAM cells. Passage 1 (p1) stocks were generated by infecting 2x10^8^ Vero-hSLAM cells with p0 at 37°C until extensive cytopathic effect (2–4 days). Cells were harvested by scraping into Opti-MEM (Gibco) and then lysed by three freeze-thaw cycles (liquid nitrogen and 37°C). Cleared lysates were aliquoted and stored in -80°C for future experiments. Viral titers were determined using the 50% tissue culture infectious dose (TCID_50_) method [60].

For passaging, p1 stocks were used to infect 1-2x10^7^ Granta-519 cells or H358 cells at MOI 0.1 for 3 days. Infected cells were collected in 1 ml opti-MEM and cell-associated MeV was released by 3 freeze-thaw cycles. In the two experiments, either 10% or 20% of the cleared lysate (100 or 200 μl) was used to infect the next dish of 1-2x10^7^ Granta-519 or H358 cells. Constant volume was used for simplicity. Because volume was standardized for passaging, the MOIs were different for each passage, with most MOI in the 0.002 to 0.2 range. Infections were carried for either 3 days or until cell lysis began. We initially attempted passaging at consistent MOI, but could not always maintain sufficient levels of infectious output, especially after host cell type switching.

### Virus titers

Either 10^6^ Granta-519, H358, or Vero-hSLAM cells were infected at the indicated MOI in triplicate. Infected cells were harvested at the indicated time points, and then lysed by three freeze-thaw cycles. Titers of cell-associated MeV were measured with the TCID_50_ method on Vero-hSLAM cells.

### Genomic RNA purification

To generate sufficient viral genomic material for sequencing, 2x10^8^ Vero hSLAM cells were infected with either 500 μl of p1 stock or half of the passaged MeV inoculum. To prevent premature cell lysis, 20 μg/ml of fusion inhibitory peptide (Z-D-Phe-Phe-OH) (Bachem California Inc., Torrance, CA) was added 24 h post infection and the infection was moved from 37°C to 32°C until harvest. Purification of MeV ribonucleocapsids (RNP) was carried out by isopycnic centrifugation as described previously [47], with one variation after pelleting of RNP through CsCl gradient [61]. The RNP pellets were solubilized in 2 ml LEH (10 mM HEPES pH 7.5, 100 mM LiCl, 1 mM EDTA) containing 1% wt/vol sodium dodecyl sulfate (SDS). RNA was extracted twice with phenol:chloroform:isoamyl alcohol (25:24:1, vol/vol/vol; Thermo Fisher Scientific; Waltham, MA) and once with chloroform:isoamyl alcohol (24:1). LiCl was added to achieve 150 mM concentration and the RNA was precipitated with two volumes 95% ethanol at -20°C overnight. RNA was resuspended in 25 μl diethyl pyrocarbonate-treated water.

### RNAseq library preparation and Illumina sequencing

RNP RNA (0.5 μg) was incubated in 10 μl of a buffered zinc solution (Thermo Fisher Scientific) for 7 minutes at 70°C, according to the manufacturer’s protocol. Fragmented RNA was purified by phenol:chloroform:isoamyl alcohol phase separation and ethanol/sodium-acetate precipitation. The concentration and integrity of the RNA was assessed on an Agilent Bioanalyzer DNA 100 chip (Agilent, Santa Clara, CA). cDNA library prep was conducted using Illumina TruSeq Stranded Total RNA Sample Prep Kit (Illumina, San Diego, CA) according to the manufacturer’s protocol. The 300 x 2 paired end sequencing of each library was performed on an Illumina MiSeq using MiSeq v2 sequencing kit and MCS v2.6.2.1 collection software. Base-calling was performed using Illumina’s RTA version 1.18.54.

### RNASeq analysis of RNP RNA

The raw BAM files from Illumina sequencing were uploaded into the Galaxy web platform [62]. We used the public server (http://usegalaxy.org/) for downstream processing and analysis of the data. Briefly, .bam files were converted into .fastq files using SamToFastq version 1.126.0, generating two FASTQ files for each data set (split by read group). Illumina adapter sequences were clipped using the FASTX-Toolkit. Low quality reads were filtered using FASTQ Quality Trimmer [63] by trimming reads from the 3’ end that had quality scores below or equal 20. Additional filtering was performed using the FASTX-Toolkit to eliminate reads that did not contain 95% or greater nucleotides having a quality score above 30. We used Bowtie2 version 2.2.6.2 [65] to process reads and align them to the *Chlorocebus sabaeus* genome (GCA_000409795.2 [64]), ribosomal RNA (hsa-45S-pre-rRNA, accession: NR_046235.3) and a MeV genome identical to IC323-EGFP (accession LC420351.1), in which the EGFP additional transcription unit sequence was either replaced with mCherry-NLS (MeV-IC323-mCherry-uN) or replaced and moved downstream of H (MeV-IC323-mCherry). IdxStats version 2.0 from the SAMTools software package [66] was run to determine read count distributions across the reference sequences. The IC323 genome aligned .bam files were then loaded in Integrative Genomics Viewer 2.3.98 (IGV; Broad Institute; Cambridge, MA) [67, 68] and aligned reads were visualized. Read count tables were generated using IGVTools [68]. Allelic frequencies were calculated and additional analyses were performed after by uploading allelic frequencies into Microsoft Excel.

### Immunoblots

Immunoblotting was performed as described previously [69]. Briefly, HeLa hSLAM cells were cultured in 6-well plates and infected with the viruses indicated. At the times indicated cells were lysed as described previously [70], incubated on ice for 30 min, and nuclei pelleted by centrifugation at 16,000 x g at 4°C for 30 min. Supernatant was collected and protein was quantified by biocinchoninic acid assay and read using the Tecan Infinite M200 Pro reader (Männedorf, Switzerland). Each lane was loaded with 20 μg total protein, fractioned by 10% SDS-PAGE, and transferred to Immobilon-P membranes (Merck; Darmstadt, Germany) using a wet transfer protocol. Membranes were blocked with 5% (wt/vol) nonfat milk (BioRad, Hercules, CA) in Tris-buffered saline (TBS), pH 6.8 for 1 h and incubated with primary antibodies at 4°C overnight. Membranes were washed three times with TBS with 0.5% (vol/vol) Tween 20 (TBST) for 5–10 min each, incubated with horseradish peroxidase (HRP)-conjugated secondary antibody at room temperature for 1h, washed three times with TBST, and incubated with Supersignal West Pico chemiluminescent substrate (Thermo Fisher Scientific). Membranes were exposed to Hyblot CL autoradiography films (Denville Scientific, Holliston, MA) or scanned using BioRad ChemiDoc Imaging System (Hercules, CA).

### Antibodies

A rabbit antiserum was raised against the peptide sequence KRNKDKPPITSGSGGAIRGIKH, corresponding to amino acids 12 to 33 of the MeV N protein, coupled to keyhole limpet hemocyanin, as described previously [71]. MeV P [70] and V [71] antisera were used at dilutions of 1:5,000. Rabbit polyclonal anti-GFP (Abcam, Cambridge, United Kingdom) was used at 1:1,000 dilution. Mouse monoclonal anti-actin (HRP) (Sigma-Aldrich, St. Louis, MO) was used at 1:25,000 dilution. Rabbit secondary antibodies conjugated with HRP were used at 1:10,000 dilution.

### Dideoxy-sequencing

Total RNA was extracted using Trizol reagent (Thermo Fisher Scientific) and precipitated with isopropanol according to the manufacturer’s instructions. Precipitated RNA was resuspended in 20 μl of DEPC-treated water and stored at -80°C. RNA (100 ng) was reverse transcribed using Superscript III reverse transcriptase (Invitrogen; Carlsbad, CA) and oligo (dT) (Promega; Madison, WI) to prime the reaction, according to the manufacturer’s protocol. PCR was performed using Phusion HF kit (New England Biolabs; Ipswich, MA) on the reverse transcribed product. For MeV-IC323-eGFP-P(-9), primers F1 5’AACCAACCATCCACTCCCAC and R 5’GAGGATCGGAAGCGTTACCT were used to amplify endogenous P; F2 5’GAGGATCGGAAGCGTTACCT and R were used for eGFP-P amplification. For amplification of P in all other viruses: 2001F 5’CTCAGCAATTGGATCAAC and P rev 5’AGGTAACGCTTCCGATCCTC [69] were used. PCRs were carried out for 35 cycles (98°C 10s, 50°C 30s, 72°C 1:20m). The PCR product was then sequenced by dideoxy methods with either 2001F or 2401F (AGAGGCAACAACTTTCC) forward primers and 2801R 5’GATTCTAGCTTGGAGATTA as a reverse primer. Chromatograms were analyzed using MEGA7 [72].

### Statistics

Student’s unpaired t-tests were performed to determine significance compared to parental or p1 viruses in growth curves and in western blots. P values are marked **, P < 0.01.

## Supporting information

S1 Fig Multiplicities of infection (MOI) of passaging experiments. (Top) MOI for the MeV-IC323-mCherry passaging experiment (related to Fig 2). p1 was used to infect Granta cells (blue diamonds) or H358 cells (green triangles) at an initial MOI of 0.1 and then 20% of the inoculum was used for each subsequent passage (2 through 14). Titers were determined for each passage, and the MOI were back calculated by dividing 20% of each passaged titer by the number of cells seeded for each infection. (Bottom) MOI for MeV-IC323-mCherry-uN passaging experiment. Passaging and MOI calculations were carried out similarly. Titers were not determined for passages L9 through L14.
(TIF)

S2 Fig RNAseq read distribution and coverage of the MeV genome. (A) RNAseq read distribution. Pie charts indicate the absolute number of MeV-specific reads and the relative coverage of MeV genomes (blue), or host cell rRNA (red), or other RNAs (green), or unmapped reads (purple). (B) Coverage plots for the MeV genome. The genome of MeV-IC323-mCherry is shown on the bottom.
(TIF)

S3 Fig Reverse strand analysis of RNA editing efficiency. mRNA sequencing using a reverse primer. (Top to bottom) RNA from HeLa-hSLAM cells infected with p1, L14, E14, or Raji-14 MeV were analyzed 48 h post infection. For a better illustration of the incidence of the +1(G) mutation, the reverse transcribed and amplified editing site-proximal P gene segment was sequenced with a reverse primer, indicated by a left-pointing arrow. The +1(G) and -10 variants are indicated by a downward arrow. Vertical dotted line: site of G-insertion. The 3G and 5A homopolymers upstream of the editing site interfere with detection of RNA editing.
(TIF)

S4 Fig The editing site-proximal mutations directly govern editing efficiency. (Top) Genome of a recombinant MeV with an editing site-proximal substitution in a GFP-tagged additional P gene copy (eGFP-P). The additional P gene was inserted downstream of the H gene. F1-R primers were used to amplify the original P gene, while F2-R primers selectively amplified the eGFP-P gene. (Bottom) Chromatograms of RNA-editing site dideoxy-sequencing after infection in HeLa-hSLAM cells 48 h post infection. An asterisk above nucleotide -9 indicates the position of the variant nucleotide. Vertical dotted line indicates the editing site. Secondary peaks downstream of the G-insertion site reflect the efficiency of RNA editing.
(TIF)

S1 Table Allelic variants (percent) above 10% in any passage of experiment 1 (related to Fig 2). (DOCX)

S2 Table Allelic variants (percent) above 10% in any passage of experiment 2 (related to Fig 5). (DOCX)
