# Supplementary figures and images for: Cyclical adaptation of measles virus quasispecies to epithelial and lymphocytic cells: To V, or not to V
Source: PLoS Pathog. 2019 Feb 15;15(2):e1007605. doi: 10.1371/journal.ppat.1007605 (PMC6395005; doi:10.1371/journal.ppat.1007605)

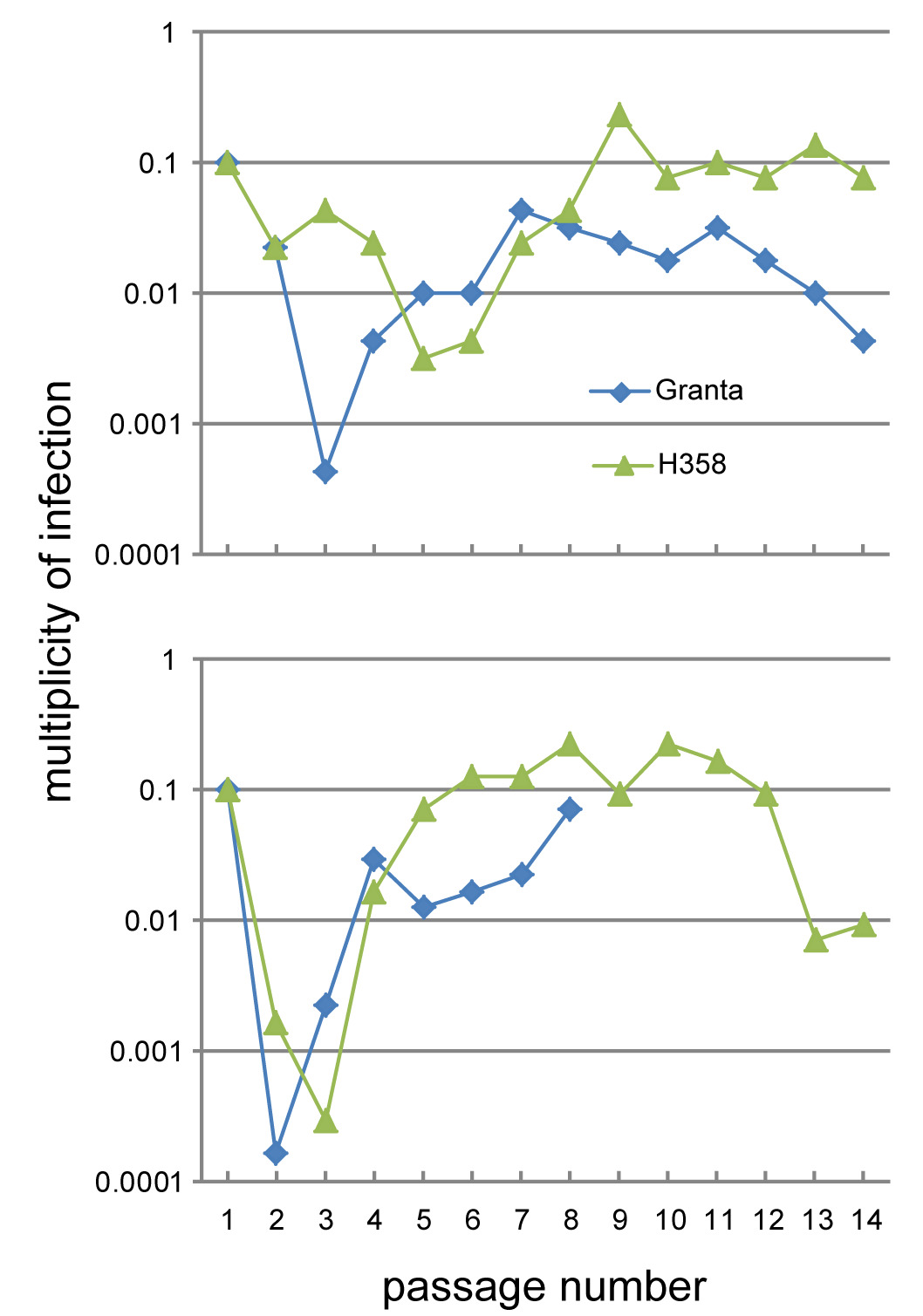

Supplement: S1 Fig — (Top) MOI for the MeV-IC323-mCherry passaging experiment (related to Fig 2). p1 was used to infect Granta cells (blue diamonds) or H358 cells (green triangles) at an initial MOI of 0.1 and then 20% of the inoculum was used for each subsequent passage (2 through 14). Titers were determined for each passage, and the MOI were back calculated by dividing 20% of each passaged titer by the number of cells seeded for each infection. (Bottom) MOI for MeV-IC323-mCherry-uN passaging experiment. Passaging and MOI calculations were carried out similarly. Titers were not determined for passages L9 through L14. (TIF) [file ppat.1007605.s001.tif]

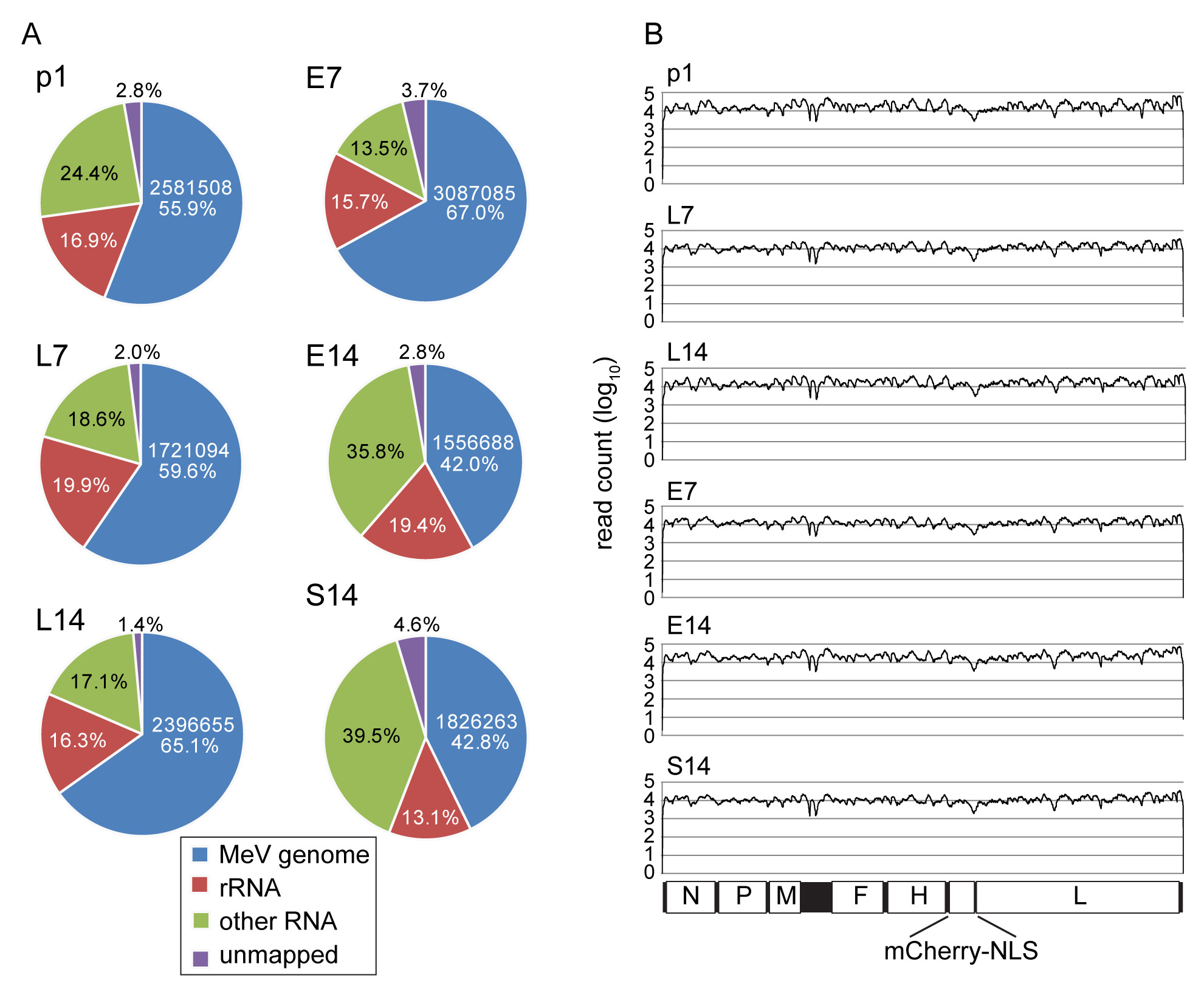

Supplement: S2 Fig — (A) RNAseq read distribution. Pie charts indicate the absolute number of MeV-specific reads and the relative coverage of MeV genomes (blue), or host cell rRNA (red), or other RNAs (green), or unmapped reads (purple). (B) Coverage plots for the MeV genome. The genome of MeV-IC323-mCherry is shown on the bottom. (TIF) [file ppat.1007605.s002.tif]

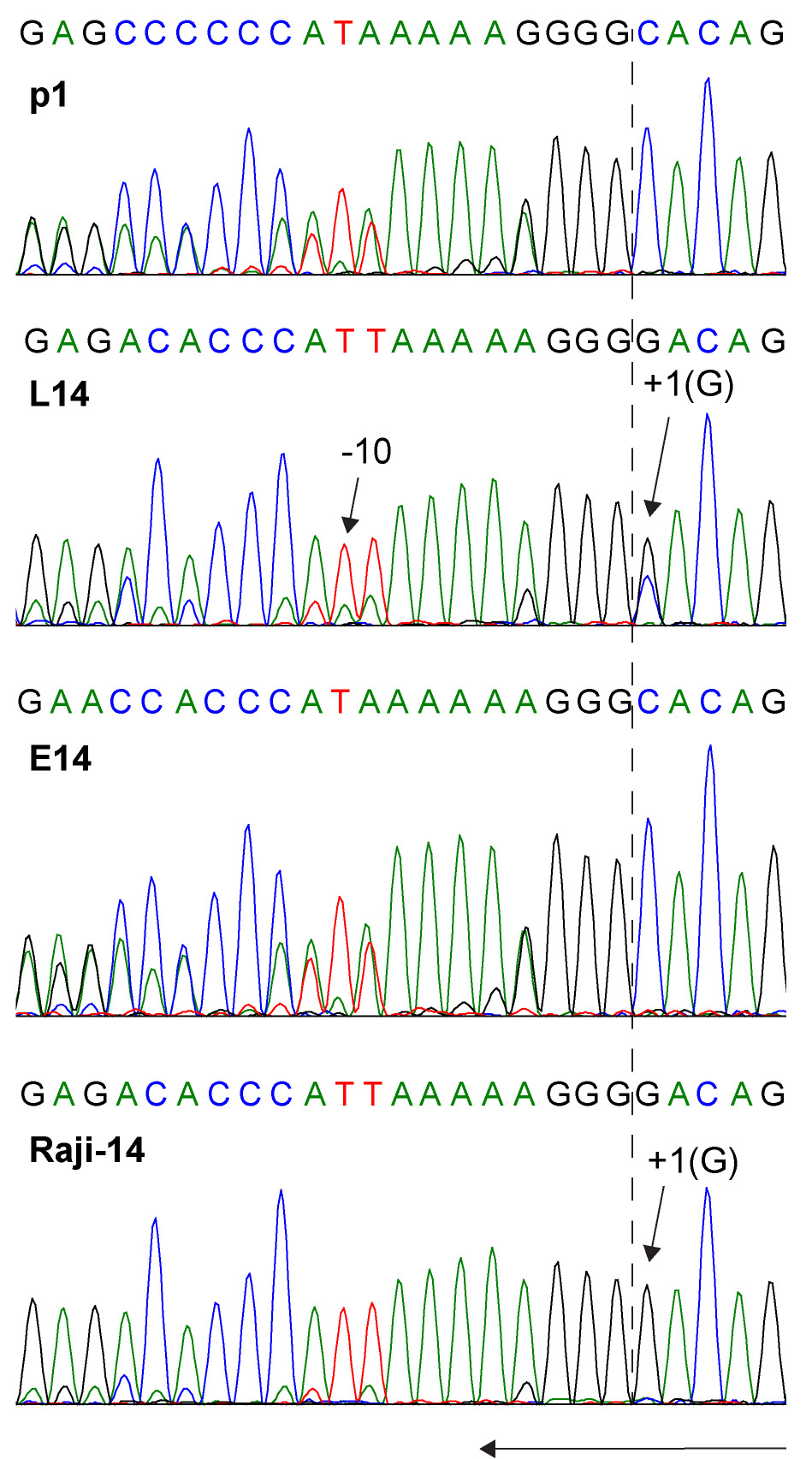

Supplement: S3 Fig — mRNA sequencing using a reverse primer. (Top to bottom) RNA from HeLa-hSLAM cells infected with p1, L14, E14, or Raji-14 MeV were analyzed 48 h post infection. For a better illustration of the incidence of the +1(G) mutation, the reverse transcribed and amplified editing site-proximal P gene segment was sequenced with a reverse primer, indicated by a left-pointing arrow. The +1(G) and -10 variants are indicated by a downward arrow. Vertical dotted line: site of G-insertion. The 3G and 5A homopolymers upstream of the editing site interfere with detection of RNA editing. (TIF) [file ppat.1007605.s003.tif]

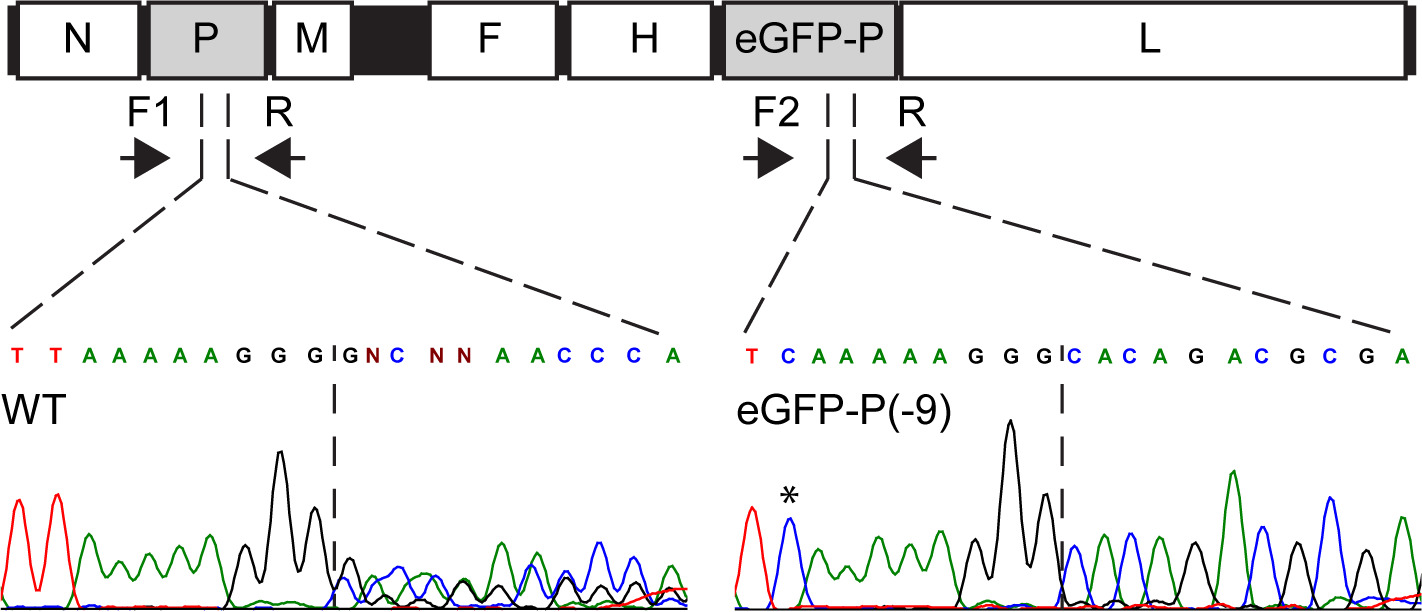

Supplement: S4 Fig — (Top) Genome of a recombinant MeV with an editing site-proximal substitution in a GFP-tagged additional P gene copy (eGFP-P). The additional P gene was inserted downstream of the H gene. F1-R primers were used to amplify the original P gene, while F2-R primers selectively amplified the eGFP-P gene. (Bottom) Chromatograms of RNA-editing site dideoxy-sequencing after infection in HeLa-hSLAM cells 48 h post infection. An asterisk above nucleotide -9 indicates the position of the variant nucleotide. Vertical dotted line indicates the editing site. Secondary peaks downstream of the G-insertion site reflect the efficiency of RNA editing. (TIF) [file ppat.1007605.s004.tif]
