# Supplementary material for: Cyclical adaptation of measles virus quasispecies to epithelial and lymphocytic cells: To V, or not to V
Source: PLoS Pathog. 2019 Feb 15;15(2):e1007605. doi: 10.1371/journal.ppat.1007605 (PMC6395005; doi:10.1371/journal.ppat.1007605)
Supplement: S1 Table — (DOCX) [file ppat.1007605.s005.docx]

**Table S1. Allelic variants (percent) above 10% in any passage of experiment 1** (related to Fig 2)

| gene | nucleotide^a^ | amino acid (protein) | passage | | | | | |
| --- | --- | --- | --- | --- | --- | --- | --- | --- |
|  |  |  | p1 | L7 | L14 | S14 | E7 | E14 |
| P | C2043T | A72V (C) |  |  |  | 11.5 |  |  |
| P | T2067C^b^ | M80T (C) | 52.8 |  |  |  |  |  |
| P | T2125C^b^ |  | 52.9 |  |  |  |  |  |
| P | T2489A^c^ | I228N (P, V) |  |  | 36.7 |  |  |  |
| P | T2490C^c^ |  |  | 29.4 |  |  |  |  |
| P | C2499G^c^ | H232D (V) |  | 53.5 | 45.6 |  |  |  |
| M | T4086C | F217L |  | 87.6 | 85.1 | 94.2 | 59.1 | 64.9 |
| M | T4275C |  |  | 43.3 | 75.6 | 14.5 | 13.2 | 13.7 |
| M | T4295C |  |  | 45.9 | 77.2 | 15.6 | 14.6 | 14.6 |
| F | C5603T | S49F |  |  |  | 23.8 |  |  |
| F | T5642C | I62T |  |  |  |  |  | 13.1 |
| F | T5750C | I98T |  |  |  |  |  | 22.3 |
| H | C7845T | T192I |  | 19.6 | 44.0 |  |  |  |
| H | A8711T^d^ | N481Y |  |  |  | 36.5 |  |  |
| H | T8847G | L526W |  |  | 48.4 |  |  |  |
| H | A9175G |  |  |  |  | 14.0 |  |  |
| mCherry | (A121G) | S26P |  |  |  | 26.1 |  |  |
| L | T10749C | S506P |  | 30.3 | 38.8 |  |  |  |
| L | A14921G^b^ |  | 20.7 |  |  |  |  |  |

1. Nucleotides are numbered as in the MeV genomic sequence, except for those within the mCherry ATU, which are numbered from its first nucleotide.
2. Variants T2067C, T2125C, and T10749C were counter-selected after 7 passages on either cell line (L7 = 0.69%, 0.72%, and 0.14%, respectively; E7 = 2.34%, 2.04%, and 0.94%, respectively) and remained at low levels after 14 passages. Since they were not detected in experiment 2, they may represent unique founder effects. We also note that mutations T2067C and T2125C appear to be linked.
3. Editing-proximal variants T2489A, T2490C, and C2499G were present in p1 at low or near-background levels (0.03%, 0.27%, and 1.32%, respectively).
4. Variant A8711T results in the H protein amino acid change N481Y, which allows binding to the CD46 receptor (J. Virol. 70, 4200-4, 1996; J. Biol. Chem. 283, 11763-71, 2008). It was positively selected only in the sequential passage series.
