# Supplementary material for: Cyclical adaptation of measles virus quasispecies to epithelial and lymphocytic cells: To V, or not to V
Source: PLoS Pathog. 2019 Feb 15;15(2):e1007605. doi: 10.1371/journal.ppat.1007605 (PMC6395005; doi:10.1371/journal.ppat.1007605)
Supplement: S2 Table — (DOCX) [file ppat.1007605.s006.docx]

**Table S2. Allelic variants (percent) above 10% in any passage of experiment 2** (related to Fig 5)

| gene | nucleotide^a^ | amino acid (protein) | passage | | | |
| --- | --- | --- | --- | --- | --- | --- |
|  |  |  | L1 | L14 | E1 | E14 |
| mCherry | (G601A) |  | 59.2 | 99.9 | 68.0 | 88.7 |
| N | T280C | L58S |  | 13.9 |  |  |
| P | G1906A^b^ | A34T (P), W26stop (C) | 10.7 |  | 24.3 |  |
| P | T2490C |  |  | 90.7 |  |  |
| M | A3475G | D13G |  |  |  | 15.0 |
| M | A3687G | T84A |  | 93.1 |  |  |
| M | G4182T | A249S |  |  |  | 33.3 |
| H | G8848T | L526F |  | 95.3 |  |  |
| L | G14145T | A1638S |  |  |  | 10.6 |

1. Nucleotides are numbered as in the MeV genomic sequence, except for those within the mCherry-NLS (mCh) ATU, which are numbered from its first nucleotide.
2. This mutation results in two different amino acid changes in the P and C proteins.
